# The differential impacts of early detection and accelerated antiretroviral therapy on the epidemiologic trend of sexually acquired HIV infection in Hong Kong

**DOI:** 10.1371/journal.pone.0274498

**Published:** 2022-09-14

**Authors:** Paul Kwok-ming Poon, Ngai-sze Wong, Wai-shing Leung, Bonnie Chun-kwan Wong, Tsz-shan Kwong, Tsz-ho Kwan, Grace Chung-yan Lui, Owen Tak-yin Tsang, Man-po Lee, Ka-hing Wong, Shui-shan Lee

**Affiliations:** 1 The Jockey Club School of Public Health and Primary Care, Faculty of Medicine, The Chinese University of Hong Kong, Hong Kong, China; 2 Stanley Ho Centre for Emerging Infectious Diseases, The Chinese University of Hong Kong, Hong Kong, China; 3 Department of Medicine and Geriatrics, Princess Margaret Hospital, Hong Kong, China; 4 Department of Health, Special Preventive Programme, Hong Kong Special Administrative Region Government, Hong Kong, China; 5 Department of Medicine, Queen Elizabeth Hospital, Hong Kong, China; 6 Department of Medicine and Therapeutics, The Chinese University of Hong Kong, Hong Kong, China; University of Washington, UNITED STATES

## Abstract

**Objectives:**

To assess impacts of early detection and prompt antiretroviral therapy (ART) on the latest epidemiologic situation to inform intervention strategy.

**Methods:**

We analysed data from two clinical cohorts in Hong Kong where sexual transmission accounted for the majority of HIV infections. The two cohorts comprised patients newly diagnosed in 2007–2008 and 2016–2018 respectively. Secular trend and differences between men who have sex with men (MSM) and heterosexual patients were examined. Predictors of late presentation (defined as CD4 ≤350 or AIDS-defining illness within 3 months of diagnosis) and prolonged interval between diagnosis and ART initiation were assessed by multivariable regressions.

**Results:**

There were 1,136 newly diagnosed HIV patients with 644 in the first and 492 in the second cohort, a majority (91.7%) presented with sexually acquired infection. There were less MSM in the first than the second cohort (50.3%% vs 87.8%, χ^2^ = 117.05, p<0.001). The mean (SD) number of days between diagnosis and ART initiation decreased from 514.3 (516.1) to 61.8 (94.2) days across the two cohorts. Younger age, non-Chinese, outpatient-based service and lower CD4 count were predictors of faster ART initiation in the first but not in the second cohort. Interval between diagnosis and ART initiation became highly uniform among groups in the second cohort. Nearly 60% were classified as late presenters in both cohorts. Heterosexuals (aOR 1.58, 95% CI 1.13–2.19) had a higher risk of late presentation.

**Conclusions:**

There was remarkable improvement in acceleration of ART initiation. Clinical implementation of accelerated ART recommendations has been effective for both MSM and heterosexuals. Late presentation was more marked among heterosexuals and remained a problem. The continued phenomenon of late presentation could offset the epidemiologic gains from accelerated ART initiation.

## Introduction

The paradigm of human immunodeficiency virus (HIV) infection management has been constantly updating over the last couple of decades, culminating in the goal of ending acquired immune deficiency syndrome (AIDS) as a public health threat by 2030 [1]. Biomedical interventions focusing on treatment as prevention (TasP) [2] have become the mainstay of public health transmission control measures. Timeliness and access of antiretroviral therapy (ART) are identified as crucial factors in achieving this goal, while the 95-95-95 cascade aspires to bring HIV treatment to all who need it [3]. Prompt initiation of ART not just improves clinical outcome but contributes towards the reduction of HIV transmission in the population. From 2015 onwards, international guidelines have unanimously recommended prompt ART initiation in all HIV patients regardless of CD4 count [4–7]. Knowingly, reduction in transmission risk by ART is optimal when it is initiated early and at a high CD4 count (>500 cell/mm^3^) [8, 9]. To achieve the best public health outcome, guidelines promulgated the good practice of reducing the time between HIV diagnosis and ART initiation [4–7]. Nevertheless, even a same-day or accelerated initiation of ART after diagnosis would not guarantee optimal clinical outcome if patients present themselves late. Early presentation and prompt treatment are therefore two inter-related criteria for ensuring the delivery of effective biomedical intervention to halt the AIDS epidemic.

Of all populations at risk of HIV transmission, men who have sex with men (MSM) have been accounting for an increasing proportion of new diagnoses in many parts of the world [10] and this key population has attracted the attention of not just researchers, but disease control professionals and policymakers. However, in order to achieve the goal of ending the epidemic in time and reducing overall community transmission, HIV spread through heterosexual activity and people who inject drugs (PWID) should not be neglected and become loophole fuelling ongoing community dissemination. Indeed, studies showed that heterosexual transmission was associated with a higher risk (odds ratios 1.73–3.77) [11–13] of late presentation as compared with MSM, and this was observed consistently in different parts of the world [14–16] including Asia [17, 18]. In addition, a recent systematic review reported that the problem of late presentation has not improved in seven countries from four continents since 2003 and until 2015 with the mean time between diagnosis and start of ART of 3.2 years [19].

Hong Kong is a metropolitan city in the Asia Pacific with the burdens of HIV/AIDS concentrated in MSM and heterosexuals and minimal caseload among PWID [20]. This bimodal nature of HIV epidemiology makes Hong Kong well positioned to study sexual transmissions and the differential impacts of TasP on the transmission of HIV. With the routine use of ART for managing HIV infection in the government-funded public service for over two decades, it is high time we assess the latest epidemiologic situation with a focus on the accelerated ART initiation and impacts of late presentation. In this study, we used real-world data from two clinical HIV treatment cohorts in Hong Kong for the analyses. The cohorts were recruited around 10 years apart with cases diagnosed before and after 2016. Our results would provide evidence on the roles of public health intervention on HIV epidemics among MSM and heterosexual patients.

## Materials and methods

### Study design and source of data

In Hong Kong, three HIV specialist clinics are providing clinical HIV/AIDS management inclusive of ART in the public healthcare sector. One of them is an outpatient day centre while the other two are hospital-based clinics. These services provide government-funded HIV care at minimal charge to local residents, the caseload of which accounted for almost all local residents diagnosed in the territory. Clinical data of patients managed at these three HIV specialist clinics constituted the anonymised HIV/AIDS cohort databases that were first established in 2006 [18]. Clinical data from two cohorts were accessed following approval. The first clinical cohort comprised HIV patients who were newly diagnosed from 2007 to 2008, with longitudinal follow-up data collected until 2012. The second clinical cohort comprised HIV patients newly diagnosed from 2016 to 2018 and followed up with longitudinal data collected until 2020.

In the two cohort databases, baseline data included gender, date of birth, ethnicity, route of transmission, intravenous drug use, ever being tested negative for HIV, date of first positive HIV test. Longitudinal CD4 count, viral load and the diagnoses of AIDS-defining illness were also included as transcribed from clinical management records at baseline and during follow-up along the treatment course. Diagnosis of all included subjects were made by a two-step approach comprising a screening enzyme-linked immunosorbent assay test and a confirmatory Western blot test for HIV antibody at reference laboratories in Hong Kong. CD4 counts enumerations were performed by a single laboratory using standard flow cytometry [21].

### Ethics and consent

The study was approved by the research ethics committees of Joint Chinese University of Hong Kong–New Territories East Cluster, Kowloon East/Central Cluster, Kowloon West Cluster, and Department of Health. Data access was approved by the Department of Health, Hong Kong Special Administrative Region Government in compliance with the Personal Data (Privacy) Ordinance. Waiver of consent was obtained (CRE2012.529) for the first cohort, whereas for the second cohort, informed written consent was obtained (CRE2015.232). This study was conducted in compliance with Declaration of Helsinki. The study period was from 2007 to 2020.

### Definitions and primary outcomes

#### Late presentation

Late presentation was defined as CD4 counts ≤350 cells/μL or development of AIDS-defining illness, regardless of CD4 cell count, within three months of HIV diagnosis taking reference from the European consensus definition [22]. It was analysed as a dichotomous variable.

#### Time interval between diagnosis and initiation of ART

Date of the first positive HIV test for diagnosis and date of start of ART were evaluated. Number of days from diagnosis to the start of ART was calculated and analysed as a continuous variable.

#### AIDS-defining illnesses

AIDS-defining illnesses were clinically diagnosed by HIV specialists in accordance with local Scientific Committee on AIDS guidelines [23] referenced from CDC’s criteria for AIDS diagnosis and reporting.

#### Time to suppressed viral load

Suppressed viral load (SVL) was defined as <200 copies HIV RNA/mL [24]. Time between the initiation of ART to SVL was calculated.

### Statistical analyses

Patients were grouped into either the “first” or “second” cohort based on year of diagnosis and a variable “cohort” was assigned and analysed as a dichotomous variable. The secular trend of the prevalence of late presentation and interval between diagnosis and initiation of ART were examined. We compared the differences in proportion of late presentation, mean number of days from diagnosis to start of ART and from start of ART to SVL between MSM and heterosexuals in the first and second cohort, using independent sample t-test and Pearson’s chi-square test. To further delineate the trend and predictors, we conducted multivariable linear and logistic regression analyses to adjust for potential confounders. Time-to-event traits are often non-normally distributed and include censored observations. In our study, the outcome variable was the interval between diagnosis and initiation of ART that was continuous and reasonably normally distributed. There were no censored observations in the data. Therefore, linear regression, rather than Cox regression, was employed to explain the relationship between one continuous dependent variable and multiple independent variables. We investigated effects of age, sex, clinic type, ethnicity, route of transmission, ever tested negative and baseline CD4 count as predictors in each cohort and across the two cohorts. Exploratory analyses of effect modification of route of transmission on the cohort effect on late presentation were also conducted. Late presentation was also investigated as a predictor of the time between diagnosis and initiation of ART. To minimise the possibility of overfitting in the multivariate logistic regression model, we adopted a forward stepwise method (probability for stepwise: entry p<0.05, removal p>0.1) to examine independent predictors. We also investigated the impact of accelerated ART initiation on the time to achieving SVL. We set the two-sided significance level at 5% and analysed using IBM SPSS Statistics for Windows, version 26 (IBM Corp., Armonk, N.Y., USA).

## Results

A total of 1,136 HIV patients were included with 644 and 492 belonging to the first (diagnoses 2007–2008) and second cohort (diagnosis 2016–2018) with a mean age at diagnosis of 38.3 and 34.9 years respectively (t = 4.94, p<0.001). Overall, sexual transmission was the route of HIV infection in a majority of cases (91.7%). The general characteristics of the two cohorts were however different (Table 1). A lower proportion of patients in the first cohort were ethnic Chinese versus that of the second cohort (73.8% vs 95.7%, χ^2^ = 96.69, p<0.001). Male accounted for 85.7% of the patients in the first cohort and 96.7% in the second (χ^2^ = 39.46, p<0.001). Fewer patients in the first than the second cohort were MSM (50.3% vs 87.8%, χ^2^ = 117.05, p<0.001) while PWID accounted for a small proportion (6.2%) of patients in both cohorts, which was higher in the first than the second cohort (0.6% vs 10.4%, χ^2^ = 46.26, p<0.001). The mean CD4 count of patients of the first cohort at diagnosis was 267 cell/μL compared to 310 cell/μL in the second cohort (t = -3.35, p = 0.001). The proportion with AIDS-defining illnesses at diagnosis or baseline CD4 count <200 cell/μL was higher in the in the first than the second cohort respectively (43.0% vs 30.1%, χ^2^ = 19.91, p<0.001). The most commonly reported AIDS-defining illness was *Pneumocystis jirovecii* pneumonia (PCP) for both cohorts (Table 1).

**Table 1:** Characteristics of newly diagnosed HIV patients in the first (years of diagnoses 2007–2008) and second cohort (years of diagnoses 2016–2018).

Variable	First cohort^†^ (n = 644)	Second cohort^†^ (n = 492)	p-value
**Demographics**			
Year of diagnosis	2007–2008	2016–2018	-
Age at diagnosis	38.3 (12.2)	34.9 (11.6)	<0.001
Male	552 (85.7%)	476 (96.7%)	<0.001
Chinese ethnicity	475 (73.8%)	471 (95.7%)	<0.001
**Route of transmission**			
MSM	324 (50.3%)	432 (87.8%)	<0.001
Heterosexual	233 (36.2%)	53 (10.8%)	<0.001
IV drug use	67 (10.4%)	3 (0.6%)	<0.001
Blood transfusion	4 (0.6%)	0 (0.0%)	-
Unknown	16 (2.5%)	4 (0.8%)	0.33
**Clinical status**			
CD4 count at diagnosis	267.2 (229.2)	310.4 (191.6)	0.001
Nadir CD4 count	187.3 (172.0)	297.3 (175.5)	<0.001
Viral load at diagnosis	224278 (389545)	281524 (643629)	0.08
Baseline CD4 count <200 cell/μL	284 (44.1%)	144 (29.3%)	<0.05
AIDS at diagnosis^‡^	74 (11.5%)	30 (6.1%)	<0.05
**AIDS Defining illnesses**			<0.001
***Pneumocystis jirovecii*** pneumonia	84 (13.0%)	33 (6.7%)	
***Mycobacterium tuberculosis*** infections	77 (12.0%)	8 (1.6%)	
Cytomegalovirus infections	37 (5.7%)	12 (2.4%)	
Penicilliosis	17 (2.6%)	5 (1.0%)	
Non-tuberculosis mycobacterial infections	15 (2.3%)	0 (0%)	
Oesophageal candidiasis	9 (1.4%)	5 (1.0%)	
Lymphoma	9 (1.4%)	0 (0%)	
Kaposi’s sarcoma	7 (1.1%)	2 (0.4%)	
Toxoplasmosis	5 (0.8%)	1 (0.2%)	
Cryptococcosis	3 (0.5%)	1 (0.2%)	
Others^§^	6 (0.9%)	6 (1.2%)	
Suspected location of HIV transmission^¶^			
Hong Kong	-	401 (81.5%)	-
China (incl. Macau)	-	52 (10.6%)	-
Southeast Asia	-	13 (2.6%)	-
Europe/North America	-	6 (1.2%)	-
Australia/New Zealand	-	2 (0.4%)	-
Others	-	8 (1.6%)	-
Unknown	-	10 (2.0%)	-

^†^ Mean value is shown with standard deviation in parentheses. For categorical variables, number of observations is shown with proportion in parentheses.

^‡^ Presence of AIDS-defining illness at diagnosis

^§^ Others include: HIV wasting syndrome, cryptosporidiosis, herpes simplex infection, lymphadenopathy, histoplasmosis, HIV encephalopathy, progressive multifocal leukoencephalopathy, salmonella septicaemia

^¶^ Information on suspected place of contact are not available in the first cohort

### Time between diagnosis and ART initiation

**Fig 1:**
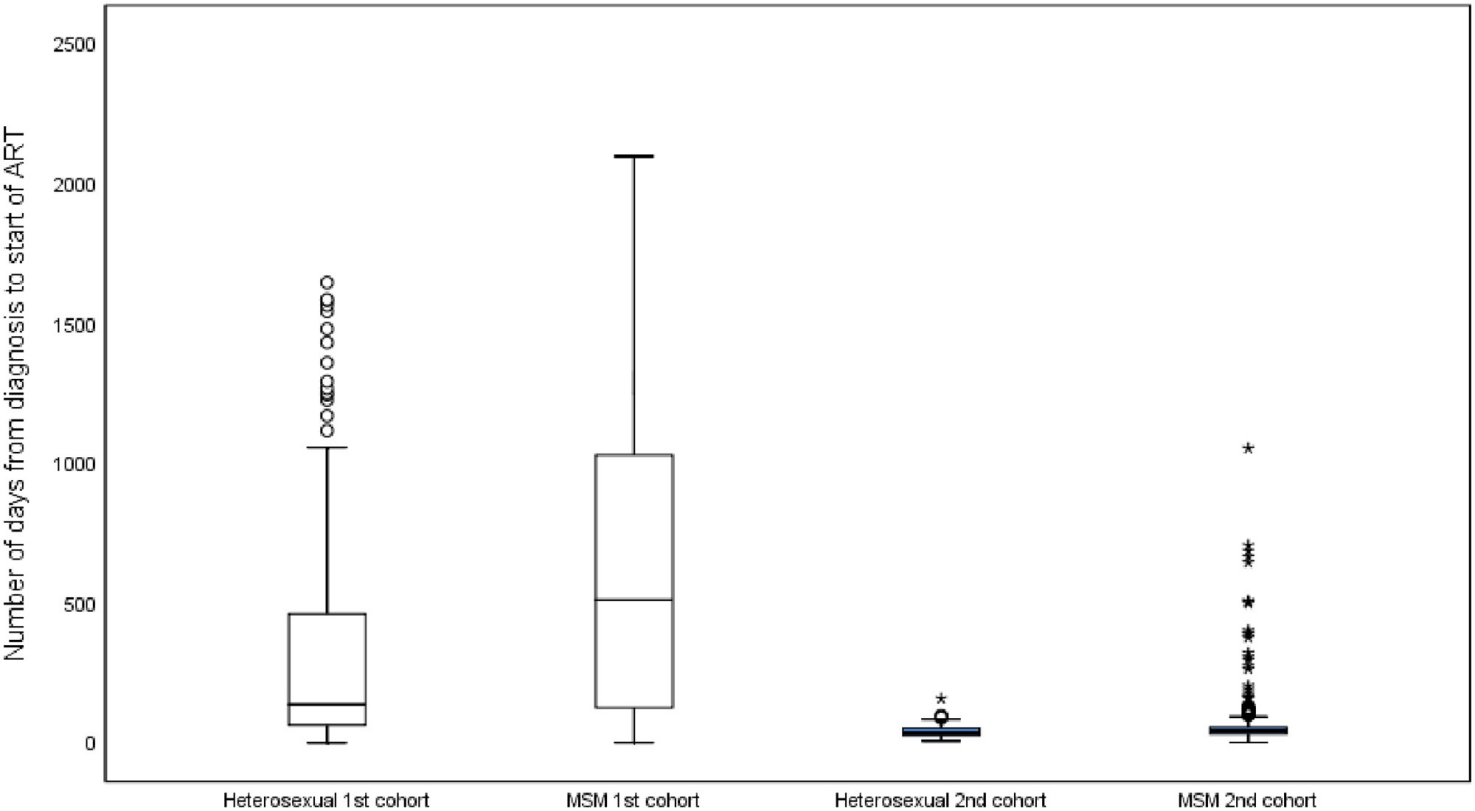
Number of days from diagnosis to ART initiation by route of transmission and between cohorts. (A) First cohort: HIV diagnosis 2007–2008; second cohort: HIV diagnosis 2016–2018. (B) ART-antiretroviral therapy; MSM-Men who have sex with men.

Overall, the number of days between the first positive HIV test and start of ART decreased markedly from a mean of 514.3 days in the first cohort to 61.8 days in the second cohort (t = 18.92, p<0.001). Classified by sexual behaviour, the interval decreased from 339.5 to 46.6 days in heterosexuals (t = 4.85, p<0.001) as compared to 628.5 to 63.6 days among MSM (t = 21.44, p<0.001) (Fig 1: Number of days from diagnosis to ART initiation by route of transmission and between cohorts). Significant difference was shown between MSM and heterosexuals in the first cohort (t = -6.08, p<0.001) but not in the second cohort (t = -1.24, p = 0.214). The mean CD4 counts at diagnosis were 312 cell/μL for MSM and 198 cell/μL for heterosexuals in the first cohort (t = -5.58, p<0.001), and 320 cell/μL for MSM and 233 cell/μL for heterosexuals in the second cohort (t = -3.16, p = 0.002).

In the first cohort, multivariable linear regression model (R^2^: 0.578) showed that younger age at diagnosis, Chinese ethnicity, outpatient-based service and high CD4 count were the independent variables associated with time between diagnosis and initiation of ART (Table 2). In the second cohort, the interval between diagnosis and ART initiation was highly uniform (Fig 1) and multivariable linear regression model developed with the available factors failed to explain variation in the outcome (R^2^: 0.038).

**Table 2:** Factors associated with number of days between diagnosis and antiretroviral therapy initiation of MSM and heterosexual patients in multivariable linear regression models.

*Variables*	*Characteristics*	*Standardized Coefficients*	*p-value*	*R* ^*2*^
**First cohort** ^†^				*0*.*578*
CD4 count at diagnosis	continuous,	0.71	<0.001	
	unit = cell/μL			
Chinese ethnicity	dichotomous,	0.121	<0.001	
	0 = non-Chinese; 1 = Chinese			
Hospital-based clinic	dichotomous,	-0.102	0.002	
	0 = outpatient-based; 1 = hospital-based			
Older age at diagnosis	dichotomous,	-0.082	0.012	
	0 = age<35; 1 = age≥35			
**Second cohort** ^‡^				0.038
*Late presentation*	dichotomous,	*-0*.*254*	*<0*.*001*	
	0 = not late presenter; 1 = late presenter			
*CD4 count at diagnosis*	continuous,	*-0*.*169*	*0*.*01*	
	unit = cell/μL			
*Ever tested negative*	dichotomous,	*0*.*103*	*0*.*027*	
	0 = never tested negative; 1 = ever tested negative			

^†^ forward stepwise method (entry p<0.05, removal p>0.1), variables excluded: sex, route of transmission, ever tested negative and late presentation

^‡^ forward stepwise method (entry p<0.05, removal p>0.1), variables excluded: sex, Chinese ethnicity, route of transmission, older age at diagnosis, hospital-based clinic and late presentation

The time from ART initiation to SVL also decreased from 169.0 days to 95.1 days from the first to second cohort (p<0.001). Similar to the interval between diagnosis and ART initiation, significant difference of time to SVL was observed between MSM and heterosexuals in the first cohort (p<0.001) but not in the second cohort (p = 0.523) (Table 3).

**Table 3:** Analysis of number of days from antiretroviral therapy initiation to achieving suppressed viral load by cohort and route of transmission.

	*Time from ART initiation to SVL* ^†^	*p-value*
Overall		
First cohort	169.0 days	<0.001
Second cohort	95.1 days	-
First cohort		
Heterosexuals	221.4 days	<0.001
MSM	137.9 days	-
Second cohort		
Heterosexuals	101.8 days	0.523
MSM	94.2 days	-

^†^Mean number of days

Abbreviations: ART, antiretroviral therapy; SVL, suppressed viral load; MSM, men who have sex with men

### Late presentation

Based on the adopted definition and among the MSM and heterosexual patients, overall 607 patients could be classified as late presenters– 57.6% and 59.0% in the first and second cohort respectively (χ^2^ = 0.19, p = 0.66). Among heterosexuals, the proportion of late presentation increased from 66.5% in the first to 73.6% in the second cohort (χ^2^ = 0.98, p = 0.32) and while among MSM, it increased from 51.2% to 57.2% (χ^2^ = 2.63, p = 0.10), the difference of which was statistically significant. Overall, there was a significantly higher percentage of late presenters among heterosexuals than MSM with a difference of 15.3% in the first and 16.4% in the second cohort. The adjusted odds ratio for being a late presenter was 1.58 (95% CI 1.13–2.19) for heterosexuals (Table 4) and the cohort effect did not modify the association. In the multivariable logistic regression model, attendance at hospital-based clinics (aOR 2.20, 95% CI 1.56–3.08), older age (≥35 years) at diagnosis (aOR 1.93, 95% CI 1.48–2.53), never tested HIV negative prior to diagnosis (aOR 1.85, 95% CI 1.34–2.56), belonging to the second cohort (aOR 1.47, 95% CI 1.07–2.01), and heterosexual transmission route (aOR 1.58, 95% CI 1.13–2.19) were associated with a higher risk of late presentation (Table 4). The interaction term cohort x route of transmission was not found to be a significant factor. The percentage of late presentation was significantly higher in heterosexuals than MSM in both the first (χ^2^ = 12.97, p<0.001) and the second cohort (χ^2^ = 5.25, p = 0.022).

**Table 4:** Factors associated with late presentation of MSM and heterosexual patients in multivariable logistic regression model^†^.

*Explanatory variables*	*aOR*	*95% confidence interval*
Clinic type		
Outpatient-based service	ref.	-
Hospital-based clinics	2.20	1.56–3.08
Age ^‡^		
Older age at diagnosis (≥35 years)	1.93	1.48–2.53
Ever tested negative		
Never tested negative	1.85	1.34–2.56
Cohort		
First cohort (diagnoses years 2007–2008)	ref.	-
Second cohort (diagnoses years 2016–2018)	1.47	1.07–2.01
Route of transmission		
MSM	ref.	-
Heterosexual sex	1.58	1.13–2.19

^†^ Forward stepwise method (entry p<0.05, removal p>0.1), variables excluded: ethnicity, interaction term “cohort x route of transmission”

^‡^ Age groups (<35 years and ≥35 years) were created

## Discussion

As demonstrated from our data analyses on two cohorts of newly diagnosed HIV infections in Hong Kong, the epidemiology of HIV infections has changed significantly over the past decade. While sexual transmission continued to prevail, there was an increasing proportion of MSM and increasing male preponderance in the recent years compared to over ten years previously. The mean age at diagnosis became 3.4 years younger in around 10 years’ time, reflecting the increase in young MSM among PLWHA. The trends of the changing epidemiology of HIV in Hong Kong were largely consistent with that observed in other parts of the world [10] but the preponderance of MSM among newly diagnosed cases in our data was even more striking than most other places. Unlike the pattern in South East Asia, PWID have never accounted for a significant proportion of HIV infections in Hong Kong, which could be attributed to the implementation of longstanding substitution treatment resulting in the achievement of harm reduction among drug users [25]. The changing epidemiology calls for enhancement of HIV combination preventive efforts towards MSM through pre-exposure prophylaxis (PrEP), another dimension of biomedical HIV prevention [26]. It must be cautioned, however, that the needs of people with high-risk heterosexual behaviours should not be ignored. In our study, heterosexually acquired infections continued to account for a smaller but substantial proportion of newly diagnosed infections, the neglect of whom could adversely affect the outcome of the city’s overall strategy on HIV prevention and control.

A remarkable overall improvement in HIV care in the last decade was the shortening of the interval by 452.5 days between diagnosis and initiation of ART in the second cohort as compared with the first. In other words, ART was initiated more rapidly for PLWHA diagnosed in 2016–2018 as compared with those diagnosed around a decade earlier with >85% improvement in terms of number of days. Effective implementation of the updated recommendations on rapid initiation of ART in clinical practices is believed to be the most important contributing factor. This should result in improved clinical outcome as well as achieving the goal of TasP. While several factors including age at diagnosis, ethnicity, clinic type and CD4 count were found to affect time between diagnosis and ART initiation in the first cohort, these factors were no longer influential in the second cohort and the difference between MSM and heterosexuals also became insignificant. This phenomenon reflected undifferentiated and equitable ART initiation among different groups of patients. In parallel there were also an overall 44% reduction in the time from ART initiation to SVL which would be crucial for the success of TaSP [2], echoing also the rationale for the U = U campaign advocated by the Joint United Nations Programme on HIV/AIDS (UNAIDS). Nevertheless, even in the more recent second cohort, the average time between diagnosis and ART initiation was still suboptimal (i.e. 61.8 days) considering the latest recommendation on early ART initiation [4–7]. In other words, there are still rooms for further improvement.

The achievement of HIV prevention through targeting PLWHA could succeed only if timely diagnosis and treatment is implemented with broad coverage. Using 350 cells/μL as the cut-off, our results showed that there was no improvement in the reduction of late presentation as over half of the newly diagnosed patients were classified as late presenters. While the identity of belonging to the second cohort was found to be an independent risk factor for late presentation, this should be interpreted with caution as the 350 cell/uL cut-off was actually higher than the mean baseline CD4 count of both cohorts. Direct comparison of the percentage of late presenters between the two cohorts (at 57.6% and 59.0% respectively) did not show any statistically significant difference. The phenomenon of late presentation was observed in other parts of the world [15, 19] and one possible reason could be the persistent lack of awareness and stigmatization [27, 28]. In Hong Kong where a majority of newly diagnosed HIV infection were sexually acquired, the problem of late presentation was higher among heterosexuals compared to MSM. These results are consistent with earlier findings in other Asian countries with similar healthcare and economic development [17, 29]. While various promoting and facilitating measures are in place for HIV-testing of MSM [30], it is important to ensure that HIV-testing strategies for heterosexuals should not be lagging behind, as has been reported in other places including China [31]. In 2019–2020, surveys conducted on key populations in Hong Kong showed that only 34.4% male clients of female sexual worker had their last HIV tests done within the preceding 12 months as compared with 61.3% among MSM [32]. This HIV-testing factor was also reflected in our results that “never tested negative” was an independent risk factor for late presentation. Older age at diagnosis was another commonly identified risk factor in other studies [15, 17]. In Hong Kong, HIV patients presenting with opportunistic infections or AIDS-defining illnesses were largely treated in the public hospitals, the care of whom are then often continued at two hospital-based HIV specialist clinics. Understandably, these advanced disease patients would have a lower CD4 count at first presentation than non-hospitalised cases. Since the outpatient day centre took care of both ambulatory and hospital discharged cases, this gave rise to a higher average CD4 count of its cases, which explained the association between hospital-based clinics and late presentation.

We acknowledge that our study carried a number of limitations. First, the routes of transmission were self-reported and it was possible that an infected MSM might have denied his sexual orientation especially at diagnosis. As the data were collected from clinical treatment records, these should have been a more reliable source of information as compared with other means (e.g. survey). Second, the clinical cohorts only included patients who attended the three public HIV specialist clinics and could have excluded patients managed by other medical service providers in the private sector. The latter was however unlikely to account for more than a few percent of the total number of PLWHA living in Hong Kong. Nevertheless, previous data showed that the bias was likely to be small [33]. Third, while the first cohort was recruited retrospectively and was a full dataset of cases managed in all three clinics, the second cohort was recruited prospectively and might have missed some cases. Written consent was only obtained for the second cohort and the difference in recruitment methods might affect the comparability of the cohorts. In addition, as compared with data in the official annual HIV surveillance reports from 2016–2018 [34], the proportion of cases in the second cohort with CD4 count ≥200 was higher (70.8% vs 64.0%, χ^2^ test, p <0.01). This would have led to an underestimation of late presentation.

Our study revealed a remarkable improvement on the acceleration of ART initiation in the recent 10 years in Hong Kong. Sexual transmission continued to be the main mode of HIV spread while PWID accounted for an insignificant proportion overall. Our analyses based on real-world data showed that clinical implementation of the updated treatment recommendations on accelerated initiation of ART has been effective for both MSM and heterosexuals. Late presentation, on the other hand, remained a problem, which was even showing worrying sign of deterioration. These late presenters, especially among heterosexuals, could undermine the efforts of TasP in minimizing community transmission. The continued phenomenon of late presentation could offset the epidemiologic gains resulting from the implementation of accelerated ART and public health actions including improved testing strategies for heterosexuals would be an important next step towards the goal of ending the AIDS epidemic.
